# Supplementary figures and images for: Uncovering anthocyanin diversity in potato landraces (Solanum tuberosum L. Phureja) using RNA-seq
Source: PLoS One. 2022 Sep 22;17(9):e0273982. doi: 10.1371/journal.pone.0273982 (PMC9498938; doi:10.1371/journal.pone.0273982)

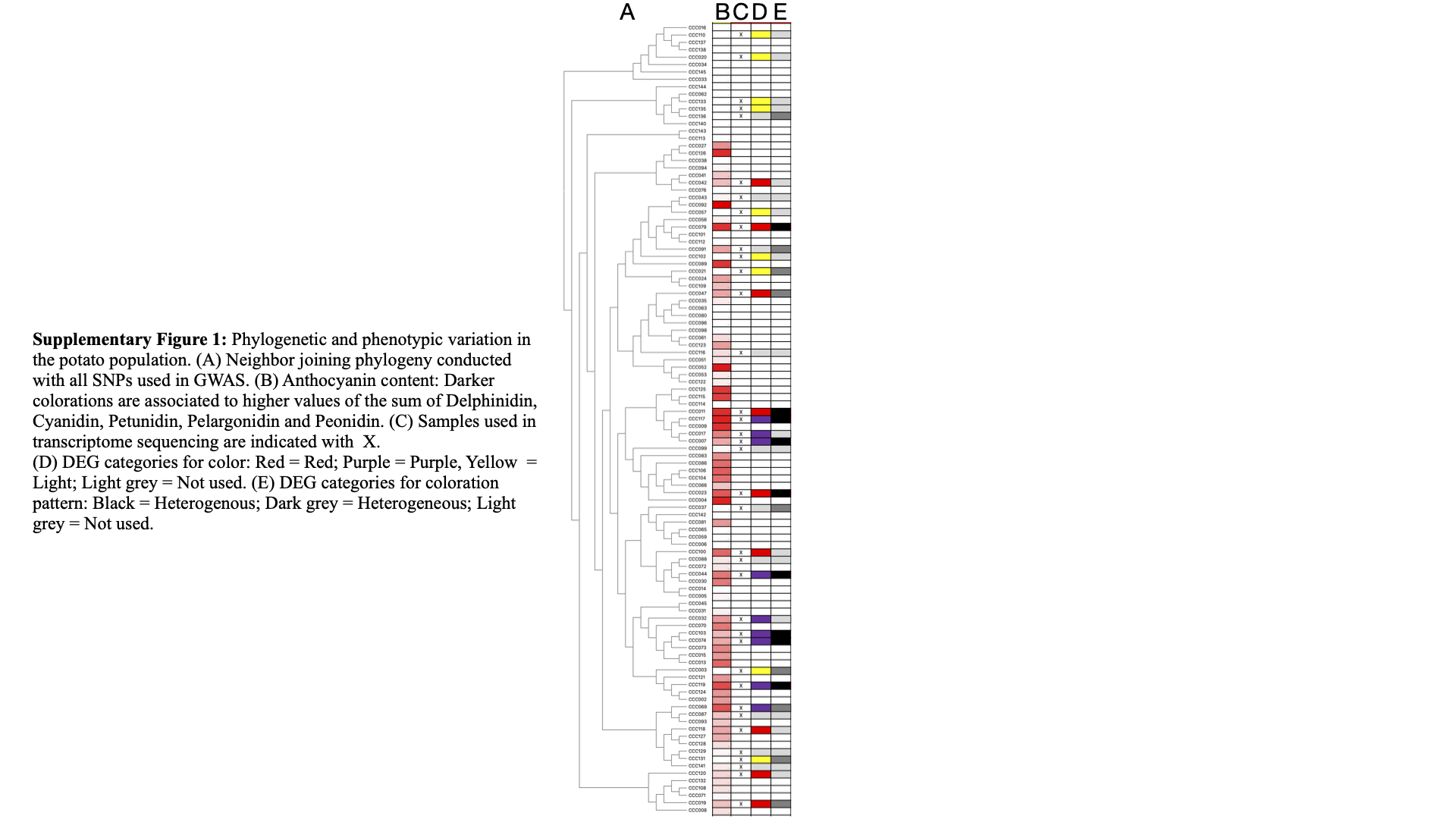

Supplement: S1 Fig — (A) Neighbor joining phylogeny conducted with all SNPs used in GWAS. (B) Anthocyanin content: Darker colorations are associated to higher values of the sum of Delphinidin, Cyanidin, Petunidin, Pelargonidin and Peonidin. (C) Samples used in transcriptome sequencing are indicated with X. (D) DEG categories for color: Red = Red; Purple = Purple, Yellow = Light; Light grey = Not used. (E) DEG categories for coloration pattern: Black = Heterogenous; Dark grey = Heterogenous; Light grey = Not used. (TIFF) [file pone.0273982.s001.tiff]

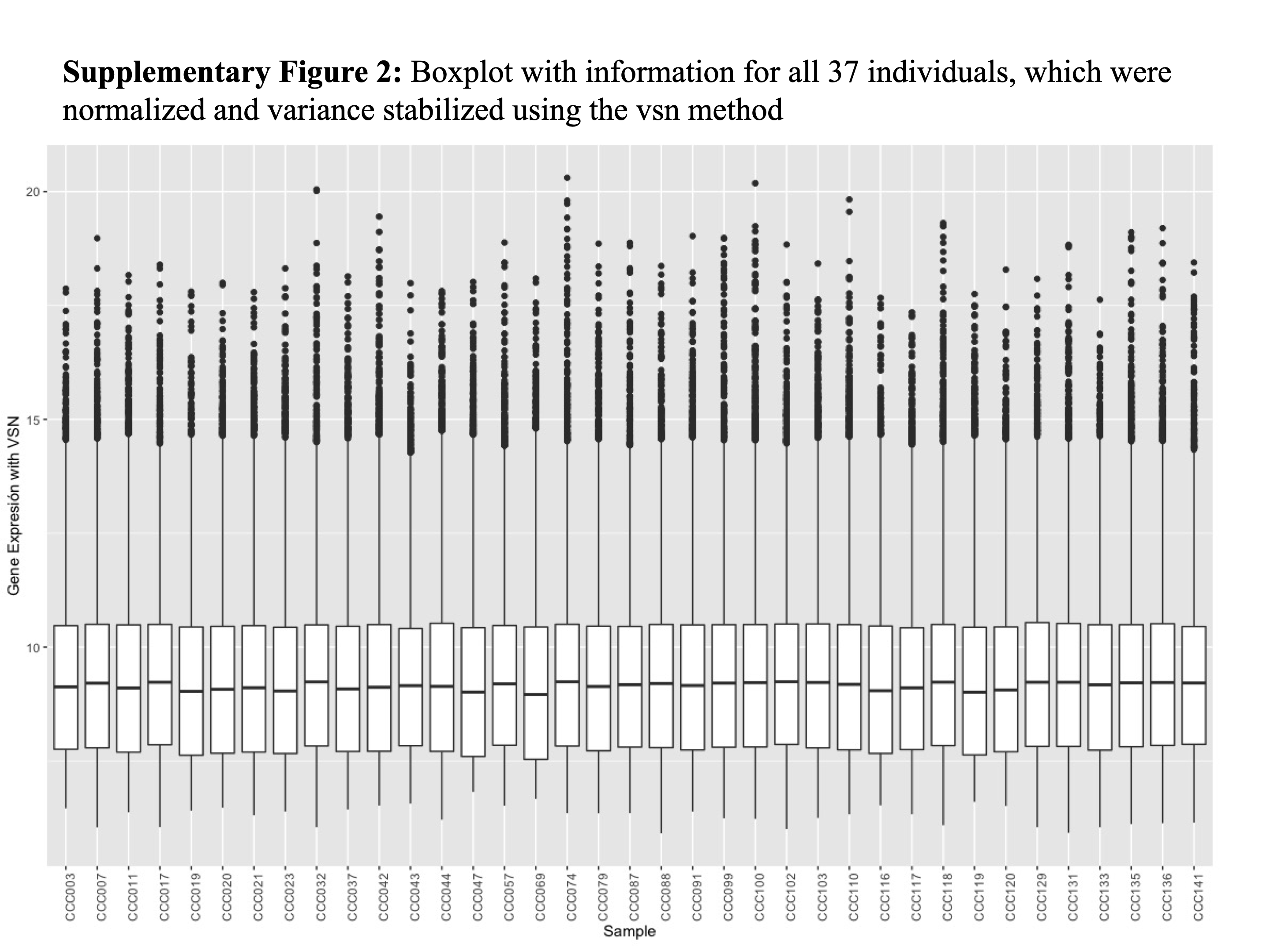

Supplement: S2 Fig — This information was normalized and variance stabilized using the vsn method. (TIFF) [file pone.0273982.s002.tiff]

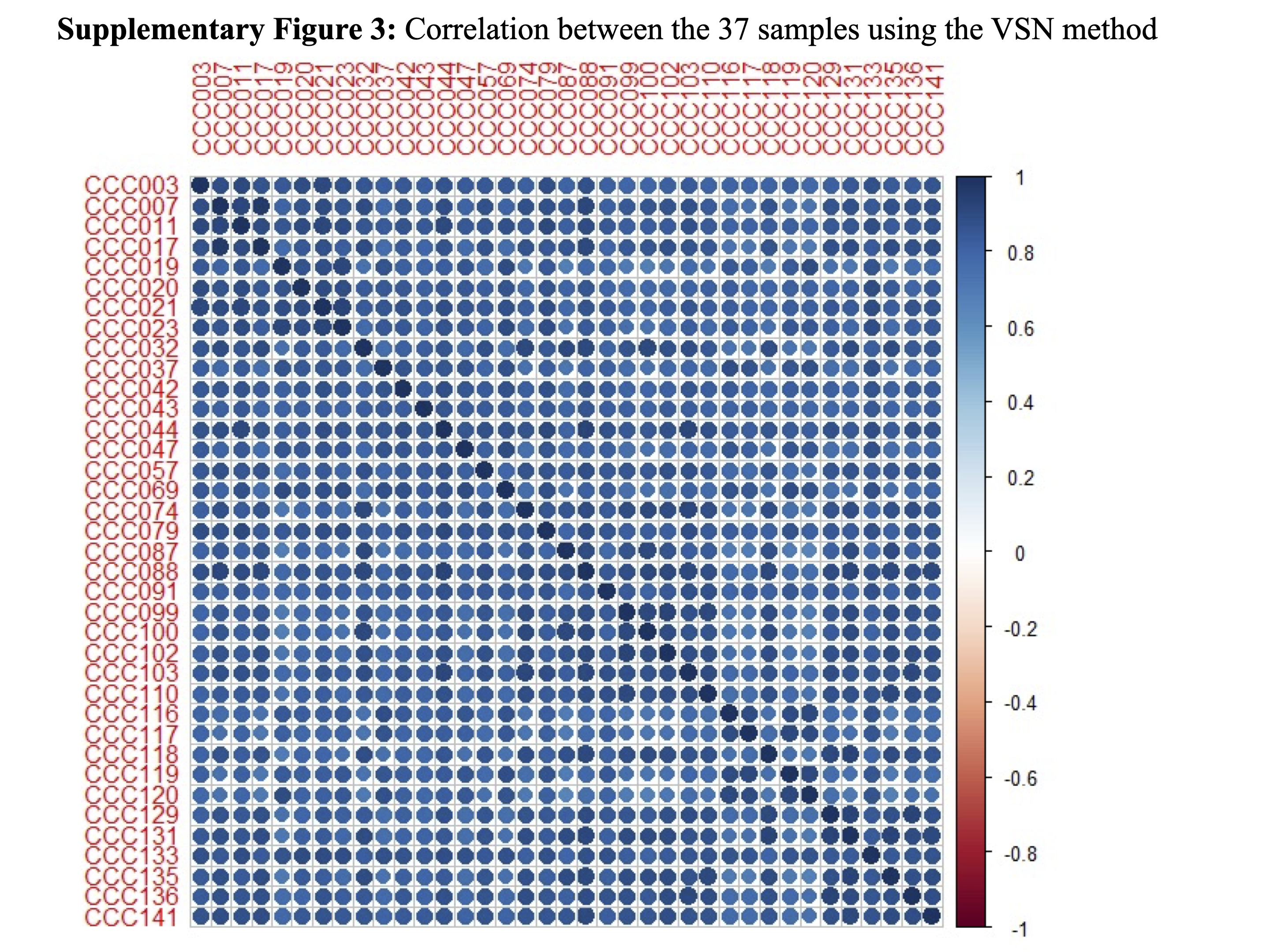

Supplement: S3 Fig — (TIFF) [file pone.0273982.s003.tiff]

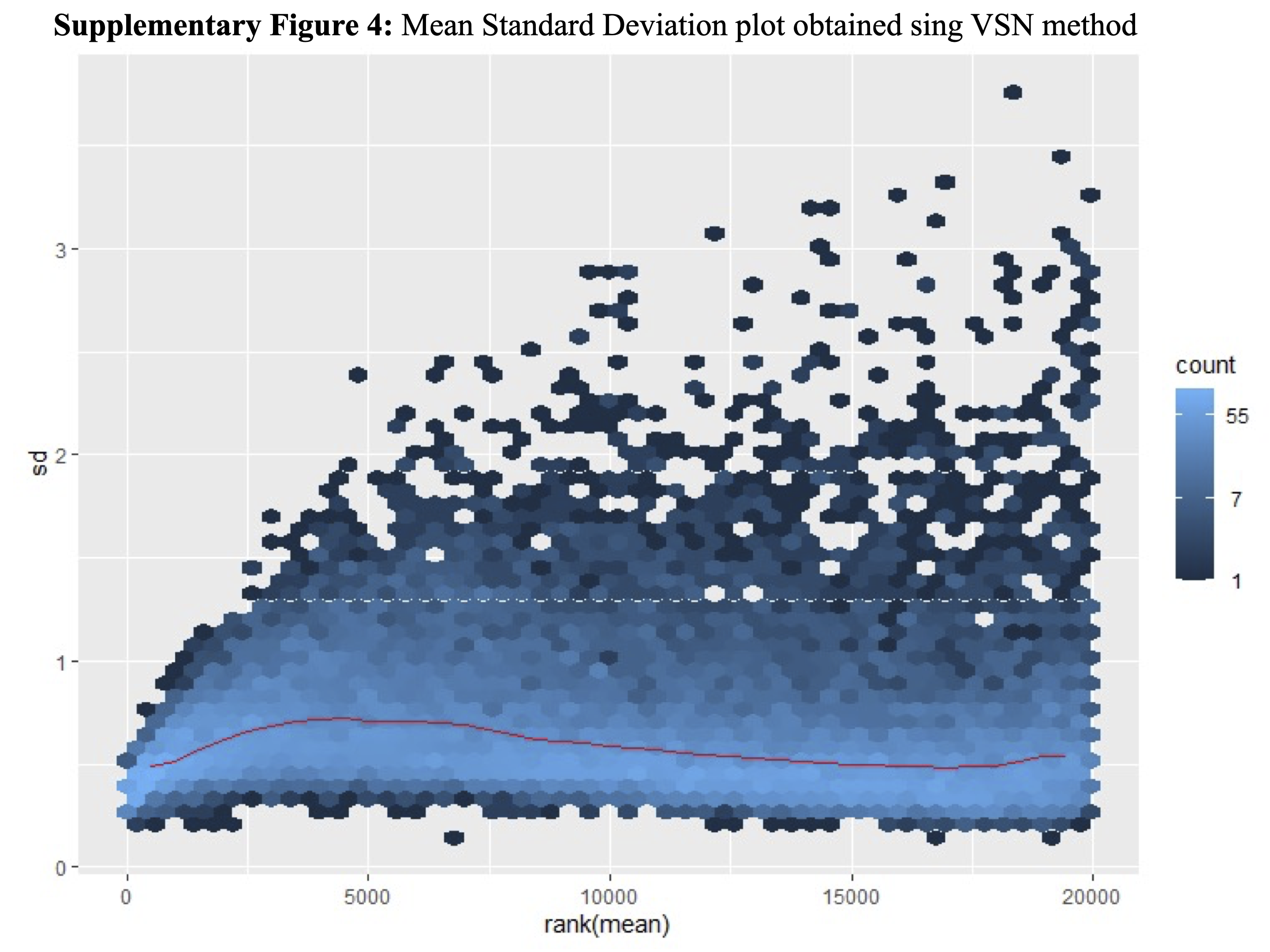

Supplement: S4 Fig — (TIFF) [file pone.0273982.s004.tiff]

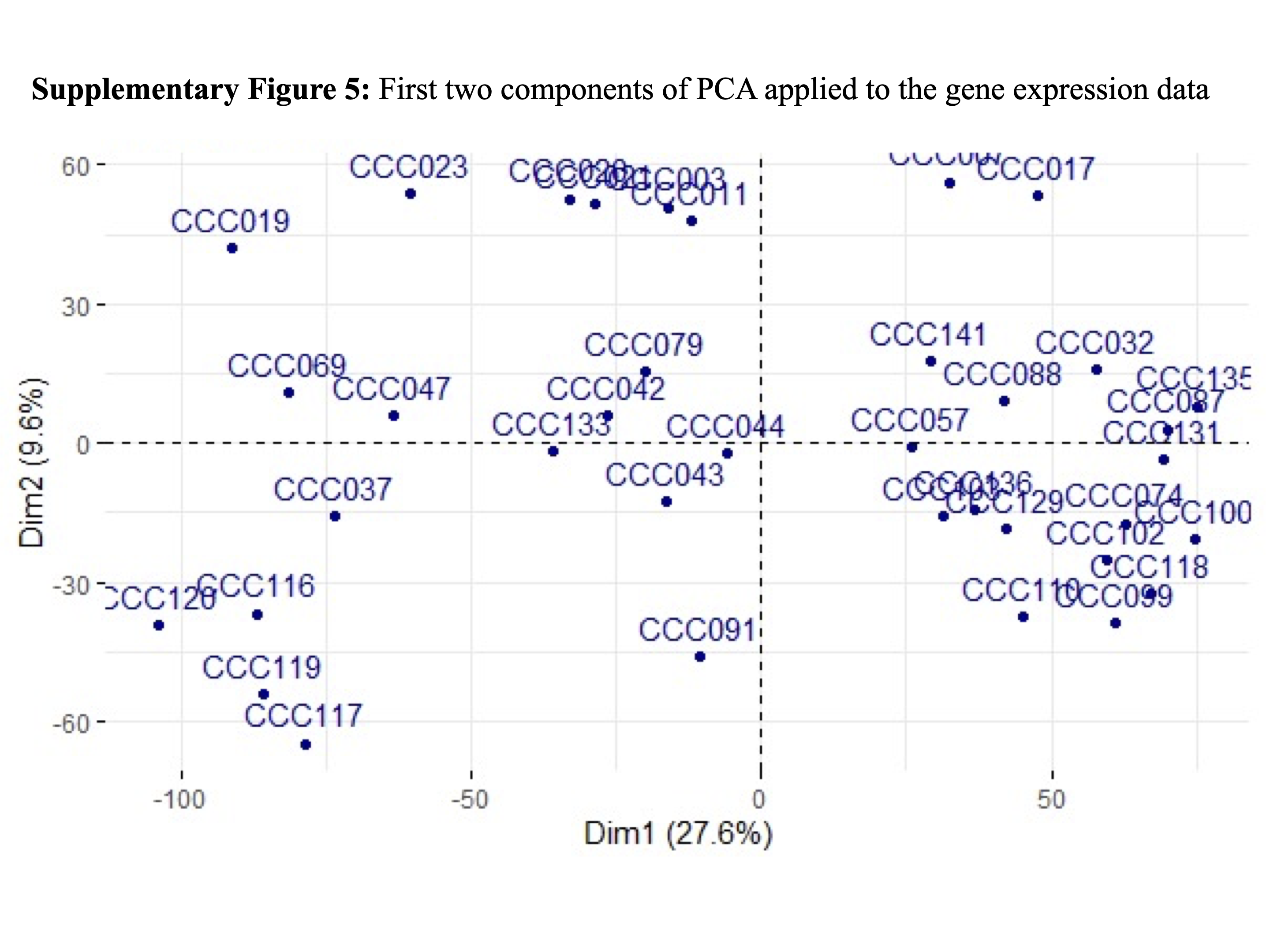

Supplement: S5 Fig — (TIFF) [file pone.0273982.s005.tiff]
