# Supplementary material for: Uncovering anthocyanin diversity in potato landraces (Solanum tuberosum L. Phureja) using RNA-seq
Source: PLoS One. 2022 Sep 22;17(9):e0273982. doi: 10.1371/journal.pone.0273982 (PMC9498938; doi:10.1371/journal.pone.0273982)
Supplement: Attachment — Submitted filename: Response_to_Reviewers_PONE-D-22-16485.docx [file pone.0273982.s013.docx]

August 12 2022

Dear

Dr. Pankaj Bhardwaj

PlosOne Academic Editor

Thank you for reviewing our manuscript entitled “Uncovering anthocyanin diversity in potato landraces (*Solanum tuberosum* L. Phureja) using RNA-seq”. The comments and suggestions from editors and reviewers helped us improve the quality of our manuscript. We hereby submit a revised version of the manuscript that addresses the points raised during the review process. In this rebuttal letter we respond to each point raised by the academic editor and reviewers. We also amended Journal requirements.

Following these reviews, we improved readability and corrected typos and other errors. We apologize for the typos and language errors present in the previous version of the manuscript. In this version of the manuscript, we carefully proofread the text and corrected all the errors highlighted by the editors. We also changed the title of the manuscript as suggested by one reviewer. We include in the submission a marked-up copy of our manuscript that highlights changes made to the original version. We include an updated cover letter.

Sincerely,

Dr. [Federico Roda Fornaguera](mailto:frodaf@unal.edu.co)

Max Planck Tandem Group Leader

Universidad Nacional de Colombia, Sede Bogotá

**Response to Journal requirements:**

1. Please ensure that your manuscript meets PLOS ONE's style requirements, including those for file naming. The PLOS ONE style templates can be found at

https://journals.plos.org/plosone/s/file?id=wjVg/PLOSOne_formatting_sample_main_body.pdf and

<https://journals.plos.org/plosone/s/file?id=ba62/PLOSOne_formatting_sample_title_authors_affiliations.pdf>.

We verified that our manuscript meets PLOS ONE's style requirements, including those for file naming.

2. Thank you for stating the following in the Acknowledgments Section of your manuscript:

We also thank the funded by the “Convenio 566 de 2014” between Universidad Nacional de Colombia and Colciecias; Colciencias Grant No 110171250437; and the project SAN Nariño number 108125-002 funded by International Deve-opment Research Centre (IDRC) and Global Affairs Canada through the Canadian International Food Security Re-search Fund (CIFSRF).

We note that you have provided funding information that is not currently declared in your Funding Statement. However, funding information should not appear in the Acknowledgments section or other areas of your manuscript. We will only publish funding information present in the Funding Statement section of the online submission form.

Please remove any funding-related text from the manuscript and let us know how you would like to update your Funding Statement. Currently, your Funding Statement reads as follows:

Funded by the “Convenio 566 de 2014” between Universidad Nacional de Colombia and Colciecias; Colciencias Grant No 110171250437; and the project SAN Nariño number 108125-002 funded by International Deveopment Re-search Centre (IDRC) and Global Affairs Canada through the Canadian International Food Security Research Fund (CIFSRF).

Please include your amended statements within your cover letter; we will change the online submission form on your behalf.

We removed any funding-related text from the manuscript. The current form of the Funding Statement does not require corrections.

3. Thank you for stating the following in your Competing Interests section:

The authors declare no conflict of interest. The funders had no role in the design of the study; in the collection, analyses, or interpretation of data; in the writing of the manuscript, or in the decision to publish the results.

Please complete your Competing Interests on the online submission form to state any Competing Interests. If you have no competing interests, please state "The authors have declared that no competing interests exist.", as detailed online in our guide for authors at http://journals.plos.org/plosone/s/submit-now

This information should be included in your cover letter; we will change the online submission form on your behalf.

We implemented the suggested change in the Conflicts of Interest section and included the required information in the cover letter.

**Detailed responses to reviewer # 1 comments:**

Other Comments for Manuscript:

1. Line 69-80, It would be better to be concise.

We made this section more concise.

2. Line 85-86, “RGCCA” instead of “Regularized and Sparse Gener-alized Canonical Correlation Analysis (RGCCA)”.

We thank the reviewer for the correction and implemented the suggested change (line 83-85).

4. Line 94, “shown in shown in the top left”, twice?

We thank the reviewer for identified the duplication in the text and corrected it (line 90).

In Fig 1.tiff, Smaller font of the name of the potato accessions would be better.

We thank the suggestion. Unfortunately, we consider that if the font was further reduced it would not be visible.

5. Line 141, Significant level for p-values is 0.01 or 0.05?

The significant level for p-value is 0,1. thank you for noticing this. We have fixed this error in the manuscript (line 121).

6. Line 227, In Fig 5, it would be better to scale in the X axis for each chromosome individually, instead of in an accumulated scale for all 12 chromosomes.

We appreciate the suggestion but respectfully disagree because we think that the suggested change would add complexity to Fig 5. Therefore, we did not implement this change.

7. Line 344, “were likely responsible for” instead of “where likely responsible for”?

We made the change and apologize for the error (line 342).

8. Line 375-397, The two sections, “Selection of material to be sequenced” and “RNA extraction and sequencing”, could be merged. Anyway, the sequencing methods could be described in a brief way.

We implemented the suggested change.

9. Line 383, 37 (even 94) accessions are not a large number of accessions, considering the sequencing cost. How the statistical analyses support “one transcriptome per accession”?

We are aware that it would have been ideal to have replicates per genotype and a larger number of sampled accession but we the statistical tests that we perform are robust to these caveats and allow us to address our research questions. Specifically, we conducted differential expression analyses using different accessions of the same coloration as biological replicates (at least 9 replicates per category). Additionally, we conducted correlation and co-expression analyses, which do not require replication per accession and work appropriately with 37 data points. We believe that this is a correct strategy to identify genes that show similar patterns across different accessions with the similar anthocyanin content, which is the goal of our study. Our goal is not to characterize or compare specific accessions and therefore we do not require replication per accession. We also believe that 37 accessions is a sufficiently big population to study the diversity of gene expression and anthocyanin content. Finally, as shown in the manuscript, most of the genes identified in our analyses have previous anthocyanin annotations, which confirms that or strategy is appropriate to identify anthocyanin genes.

We clarify this in the methods section (line 389-394).

10. The genetic backgrounds of 37 landraces are different, even though all these landraces belong to Phureja group. Did the difference interfere the Differential Expression Analyses? It should be discussed in the manuscript.

When we selected accessions with similar coloration patterns, we selected representative accessions in the different phylogenetic groups (Supplementary Figures 1 and 3). In this way we minimized the effect of population structure in our analyses.

This was clarified in the corrected version of the manuscript (line 374-378).

11. The quality of the written English is not satisfying. There are spelling and vocabulary mistakes needing corrections in the manuscript. Such as “an-thocyanin” in abstract. References in the text need to be enclosed in brackets, such as line 378.

Following the reviewer suggestion, we improved readability and corrected typos and other errors. We apologize for the typos and language errors present in the previous version of the manuscript. In this version of the manuscript, we carefully proofread the text and corrected all the errors highlighted by the editor.

12. There are two names of the MYB gene (Soltu. DM.10G020850), StAN1 and StAN2.

The code: Soltu. DM.10G020850 is just for StAN2 not for StAN1. We corrected this error.

13.The differential genes analyzed were not verified by any experiments, such as qPCR and dual luciferase reporter assay.

The goal of this study was to identify genes that showed population-level correlations between expression and anthocyanin content. Most of the candidate genes identified have already been associated to anthocyanin production in previous studies of potato cultivars. In the future we plan to validate some of the identified candidates with transgenesis and qPCR however we believe it is beyond the scope of this manuscript.

14. The WRKY13 gene in this manuscript has been reported and should be included in the discussion. StWRKY13 promotes anthocyanin biosynthesis in potato (Solanum tuberosum) tubers. Functional Plant Biology, 2022, 49(1):102-114

We appreciate this suggestion and included the suggested reference (line 609-610).

15. “our results are summarized in Fig 4” in line 167 should be deleted.

We were grateful for this recommendation and we removed this section from the paragraph (line 169).

**Detailed responses to reviewer # 2 comments:**

Reviewer #2: The manuscript “The contribution of gene expression to anthocyanin diversity in potato landraces (Solanum tuberosum L. Phureja)” is well presented. This can be accepted after addressing minor comments as below:

Title: Modify it, something like: ‘Uncover anthocyanin diversity in potato landraces applying RNA-seq.......approaches’

The title was changed to “Uncovering anthocyanin diversity in potato landraces (*Solanum tuberosum* L. Phureja) using RNA-seq”.

Abstract: Line 26, modify it, or mention first and second crop. Since there are several other vegetables rich in antioxidants like tomato etc.

The three crops that provide the highest proportion of antioxidants in the human diet are maize, tomato and potato. We have made the respective change in the manuscript.

Introduction: Line 40-41, modify, not good sentence. Modify from L40 to 64, in a story format. Its a bit scattered.

We have made the requested change and those paragraphs of the manuscript have been re-written (line 39-63).

Results: L89-90: Besides two, mention the correlation between phylogeny and anthocyanin production as well.

We are grateful for this recommendation and made the suggested correction (line 88-89).

L102: (ie spots) ?? Correct ‘i.e.’ spots

We were grateful for this recommendation and we made this correction (line 101).

Check for typo and grammatical errors in throughout the manuscript.

Following the reviewer suggestion, we improved readability and corrected typos and other errors. We apologize for the typos and language errors present in the previous version of the manuscript. In this version of the manuscript, we carefully proofread the text and corrected all the errors highlighted by the editor.
